# The influence of in vitro pectin fermentation on the human fecal microbiome

**DOI:** 10.1186/s13568-018-0629-9

**Published:** 2018-06-16

**Authors:** So-Jung Bang, Gayoung Kim, Mi Young Lim, Eun-Ji Song, Dong-Hyun Jung, Jun-Seok Kum, Young-Do Nam, Cheon-Seok Park, Dong-Ho Seo

**Affiliations:** 1 Graduate School of Biotechnology and Institute of Life Science and Resources, Kyung Hee University, Yongin, 17104 Republic of Korea; 2 Research Group of Healthcare, Korea Food Research Institute, Wanju, 55365 Republic of Korea; 3 Department of Food Biotechnology, Korea University of Science and Technology, Daejeon, 34113 Republic of Korea; 4 Research Group of Food Processing, Korea Food Research Institute, Wanju, 55365 Republic of Korea

**Keywords:** Pectin, Fecal microbiota, Fermentation, Short chain fatty acids, Prebiotic

## Abstract

Pectin is a complex dietary fiber and a prebiotic. To investigate pectin-induced changes in the gut microbiome and their effects on the short chain fatty acids (SCFAs) production, we performed in vitro pectin fermentation using the feces of three Korean donors. The pectin degradations in all three donors were observed. While the donors displayed differences in baseline gut microbiota composition, commonly increased bacteria after pectin fermentation included *Lachnospira*, *Dorea*, *Clostridium*, and *Sutterella*. Regarding SCFAs, acetate levels rapidly increased with incubation with pectin, and butyrate levels also increased after 6 h of incubation. The results suggest that pectin fermentation increases bacterial species belonging to *Clostridium* cluster XIV (*Lachnospira*, *Dorea*, and *Clostridium*), with *Lachnospira* displaying the greatest increase. The results also confirm that pectin fermentation leads to the production of acetate and butyrate.

**Electronic supplementary material:**

The online version of this article (10.1186/s13568-018-0629-9) contains supplementary material, which is available to authorized users.

## Introduction

Recent studies have demonstrated that the gut microbiota plays important roles in human health, and are associated with diseases. The pathogenic mechanisms of various diseases and disorders, such as irritable bowel syndrome (Hyland et al. 2014), Crohn’s disease (Gevers et al. 2014), and non-alcoholic steatohepatitis/non-alcoholic fatty liver disease (Icaza-Chávez 2013; Sánchez et al. 2017) are associated with the composition and diversity of the gut microbiota (Laparra and Sanz 2010). The constitution of the gut microbiota can be influenced by endogenous and environmental factors, such as one’s dietary, antibiotic, xenobiotic, and probiotic intakes (Falony et al. 2016).

Prebiotic food ingredients feed the intestinal microbiota, and can be used to selectively promote the growth of specific microbiota (Laparra and Sanz 2010). Most prebiotics are carbohydrates, such as inulin, fructooligosaccharide, and human milk oligosaccharide, which are not digested by human digestive enzymes (Bindels et al. 2015; Coppa et al. 2006). Microorganisms in the intestines produce energy and short-chain fatty acids (SCFAs) including acetate, propionate, and butyrate, affecting the host though prebiotic fermentation (Flint et al. 2012). The SCFAs produced by the gut microbiota have positive effects on immune function and ameliorate metabolic diseases such as obesity and type 2 diabetes (Clemente et al. 2012; Gerritsen et al. 2011).

Differences in intestinal microbiota composition can exist depending on diet (Conlon and Bird 2014; David et al. 2014), and in particular, on the types of nutrients ingested in various countries, environments, and cultures (Marques et al. 2010). Asian diets tend to be high in carbohydrates, while western country diets are relatively high in fat (LeCroy and Stevens 2017; Li et al. 2017). The Korean diet, one of the Asian diets, tends to be a high-vegetable diet compared with the typical western diet because of traditional foods including Kimchi (Kim et al. 2016; Song and Joung 2012; Lee et al. 2002).

Pectin is a complex polysaccharide found in the cell walls of a variety of vegetables and fruits, which is mainly composed of d-galacturonic acid (GalA) with α-(1-4) glycosidic linkages (Sriamornsak 2003). Pectin is a candidate prebiotic because it is not well degraded by human digestive enzymes, but is by microorganisms (Holloway et al. 1983). Pectin is degraded by the gut microbiota, producing SCFAs and changing the composition of the intestinal microbiota (Chung et al. 2016; Marounek and Dušková 1999). Although many studies have confirmed the degradation of pectin and the production of SCFAs by gut microbiota species of the genera *Bifidobacterium*, *Faecalibacterium*, *Anaerostipes*, and *Roseburia* (Duncan et al. 2004), very few metagenomics studies have been performed on the human gut microbiota following in vitro fermentation of pectin. In addition, no studies have been performed examining pectin utilization and gut microbiota changes in Koreans, which have high vegetable consumption. Thus, in this study, we explored the process of pectin degradation and the compositional changes in the gut microbiota of three Korean subjects after pectin fermentation.

## Materials and methods

### Donor information

All donors (males, ages 29, 30, and 30) were healthy and did not have any gastrointestinal disease. Donors had consumed a regular diet and had not received antibiotic treatment in the last 6 months. The study was approved by the Institutional Review Board of Kyung Hee University (IRB file no. KHSIRB-17-004). All experiments were performed in accordance with relevant guidelines and regulations. Informed consent was obtained from all participants.

### Fecal collection

Fecal samples (20 g) were collected from three volunteers under anaerobic conditions and transported within 1 h after excretion. Fecal samples were diluted fivefold with sterile phosphate-buffered saline in an anaerobic chamber. After mixing, the resultant fecal slurries were homogenized and immediately inoculated in the prepared medium.

### Growth media

Pectin from citrus peel was purchased from Sigma-Aldrich (St. Louis, MO, USA), and was composed of > 74% galacturonic acid. Because a basal medium is non-selective and supports the growth of several organisms, we selected a basal medium for this study; the basal medium used was the chopped meat (CM) broth containing 15% (v/v) of bovine rumen fluid (CMR). Each liter of CMR consisted of peptone (30.0 g), yeast extract (5.0 g), dipotassium phosphate (5.0 g), l-cysteine (0.5 g), and resazurin (0.001 g). Pectin was added to a final concentration of 1%. CMR medium with 1% pectin (CMRP) was stirred on a hot plate to dissolve the pectin, transferred to serum vials, and capped. Sealed CMRP medium was flushed with 99.5% CO_2_ gas and sterilized by autoclave at 121 °C for 15 min.

### Batch culture incubations

To investigate changes in microbial diversity and pectin degradability, 100 μL of prepared feces was inoculated into 20 mL of CMRP medium (baseline). Cultures were incubated at 37 °C at 150 rpm, with sampling at various incubation times (0, 6, 12, 18, 24, 36, and 48 h). All samples were immediately frozen and stored at − 72 °C. Incubations were performed in CMRP medium in triplicate.

### Determination of total sugar

The total sugar in culture was measured by the phenol–sulfuric acid method. A 5% phenol solution (200 μL) was added to 100 μL of culture supernatant from each time point. The reaction mixture was mixed with 800 μL of 99% sulfuric acid and vortexed. After cooling for 20 min at 25 °C, 250 μL of each mixture was added to a 96-well microplate. The absorbance of the phenol–sulfuric acid was measured at 550 nm using an iMark Microplate Absorbance Reader (BioRad Laboratories, Inc., Hercules, CA, USA).

### Determination of reducing sugar

The reducing sugar in culture was measured using 3,5-dinitrosalicylic acid (DNS). Culture supernatants from each time point (20 μL) were diluted in 80 μL deionized water. Reducing sugar was detected by adding 300 μL of DNS solution and boiling for 5 min. After cooling on ice, the absorbance was measured at 555 nm using an iMark Microplate Absorbance Reader.

### Thin liquid chromatography (TLC) analysis

TLC analysis was performed with TLC Silica gel 60G F_254_25 Glass plates (Merk Millipore, Billerica, MA, USA) after activating at 110 °C for 5 min. Culture supernatants from each time point were loaded onto TLC plates and placed in a TLC chamber containing a 5:2:3 (v/v/v) solvent mixture of 1-butanol:acetic acid:water for degradation product analysis. Plates were dried, rapidly soaked into 0.3% (w/v) 1-naphthol and 5% (v/v) sulfuric acid in methanol, dried again, and placed on a 110 °C oven for 10 min.

### DNA extraction and 16S rRNA gene sequencing

Fecal bacterial DNA from samples taken at various times (0, 6, 12, and 18 h) was extracted using a QIAamp DNA Stool Mini kit (Qiagen, Valencia, CA, USA), in accordance with the manufacturer’s instructions, including bead-beating twice for 5 min. The V1–V2 regions of 16S rRNA genes were amplified by polymerase chain reaction (PCR) using universal primers (8F and 338R) with barcode sequences for multiplexing sample reads. PCR was performed using a PCR Thermal Cycler Dice (Takara, Shuzu, Japan) and recombinant Taq DNA polymerase (Takara). The PCR conditions were as follows: 95 °C for 5 min; 30 cycles of 30 s at 95 °C, 1 min at 61 °C, and 40 s at 72 °C; and 5 min at 72 °C. The amplified 16S rRNA gene products were purified with an AccuPrep PCR Purification Kit (BIONEER, Daejeon, Korea).

PCR product concentrations were measured on a NanoDrop ND-1000 (NanoDrop Technologies Inc., Wilmington, DE, USA) and mixed to a constant concentration such that the total concentration was 1 mg. The Ion Xpress Plus Fragment Library Kit (Thermo Scientific, Wilmington, DE, USA) was used to form the amplicon library according to the manufacturer’s instructions, and quantification of the amplicon library was performed using a Bioanalyzer 2100 (Agilent Technologies, Inc., Santa Clara, CA, USA). The amplicon library was sequenced on an Ion Torrent PGM system (Thermo Scientific, Wilmington, DE, USA).

### Bioinformatic analysis

The quantitative insights into microbial ecology (QIIME) pipeline (Caporaso et al. 2010) was used to analyze the sequences. After quality checking the FASTQ file, the barcode sequences of the amplicons were removed. Sequences of 300–440 bp size were filtered and dimers and chimeras were removed, based on Ribosomal Database Project (RDB) database, using ultra-fast sequence analysis (USEARCH 8.1 to ensure high quality. Operational taxonomic units were analyzed based on the Greengenes 13_8 database (McDonald et al. 2012) identified with 97% similarity. Principal coordinates analysis (PCoA; based on the Bray–Curtis distance) was performed using QIIME 1.9.1. The identification of microbial taxa that were significantly associated with the incubation time was conducted using multivariate association with a linear model (Morgan et al. 2012). Associations with a Benjamini–Hochberg false discovery rate-corrected *p* value (q value) of < 0.1 were considered significant. The raw data was uploaded to NCBI sequence read archive database (accession number: DRA006695).

### SCFA concentration analysis

CMRP medium supernatants containing the fecal inoculum of the three donors at each incubation time (0, 6, 12, 18, 24, 36, and 48 h) were transferred to new tubes, and aliquots were frozen at − 20 °C for SCFA analysis. Before analysis, samples were filtered through membrane filters (pore size: 0.25 μm). SCFA analysis was performed by ion chromatography (IC) using a 940 Professional IC Vario (Methrohm, Herisau, Switzerland) composed of a two-channel peristaltic pump and a 945 Professional Detector Vario conductivity detector, with an 889 IC Sample Center (Methrohm). IC Net 3.1 software was used to record the data. Ion exclusion was performed on a Metrosep Organic Acid 250/7.8 column (Methrohm) and 0.1% sulfuric acid was used as the mobile phase, at a flow rate of 0.5 mL/min and pressure of 6.99 MPa. Samples (20 μL) were injected into columns maintained at 30 °C. The peak height, peak area, and retention time of recorded samples and acetic acid, propionic acid, butyric acid, valeric acid, and isovaleric acid standards were used to measure concentrations.

## Results

### Pectin degradation by human feces according to incubation time

**Fig. 1:**
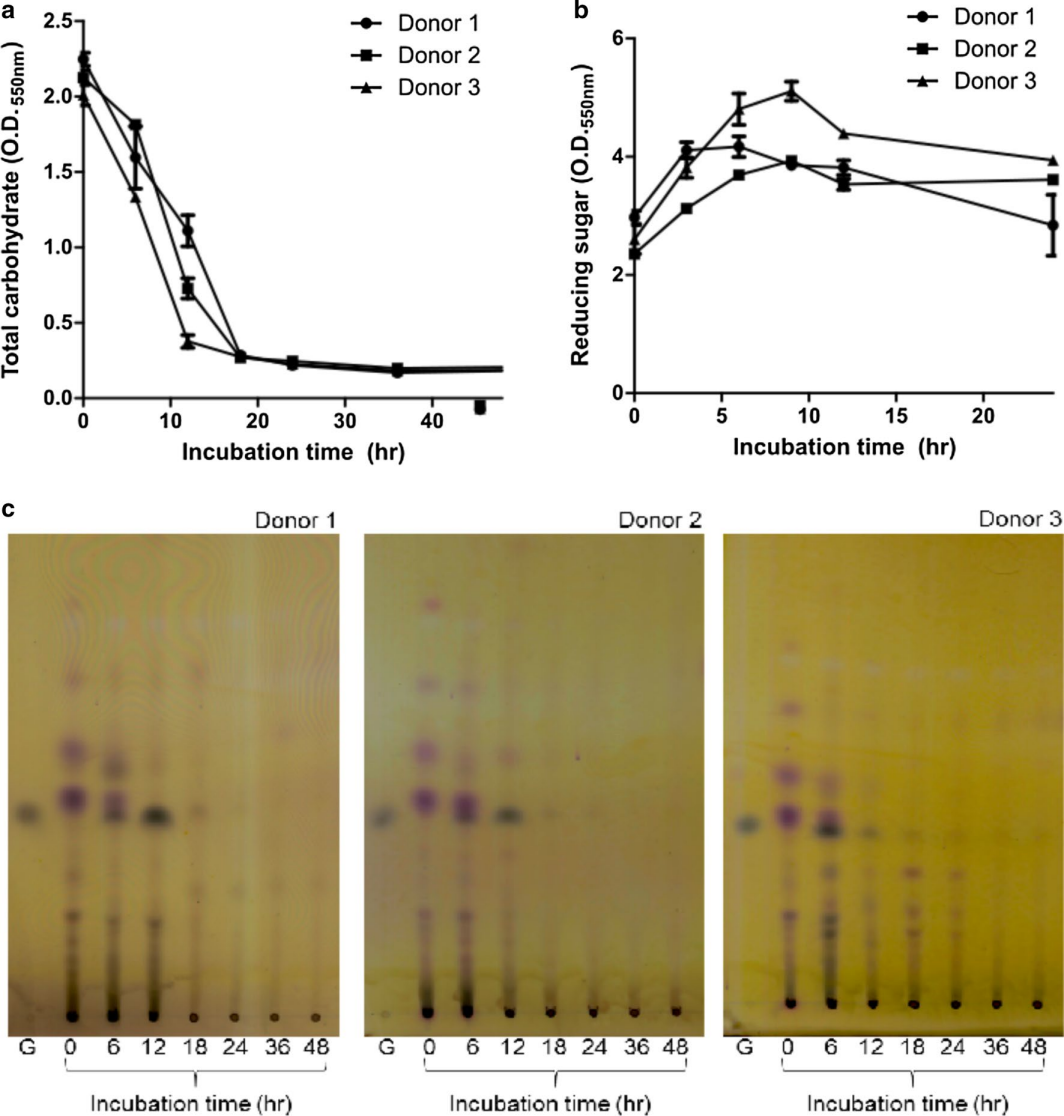
Total carbohydrates (**a**), reducing sugars (**b**), and decomposed pectin products (**c**) in each donor at various incubation times were analyzed by TLC

Three samples of feces were anaerobically cultured with pectin, and the total carbohydrates in the medium were measured every 6 h after inoculation (Fig. 1a). Almost all of the carbohydrates in the medium were consumed within 18 h in all experimental groups. These results suggest that the carbohydrate components in the medium were used by the gut microbiota present in the feces, and particularly, that the pectin was decomposed and used by the gut microbiota. There were differences in the efficiency of carbohydrate usage in each sample. The sample from donor 3 depleted the carbohydrates from the medium more rapidly than the other samples. The amount of reducing sugars was determined for each sample with increasing incubation time (Fig. 1b). The increase in reducing sugar was the highest in donor 3, consistent with the carbohydrate utilization observations. The samples from donors 2 and 3 displayed increased reducing sugars for the first 9 h, followed by decreasing levels thereafter, while the sample from donor 1 showed decreased reducing sugars in the medium from 6 h onward. This suggests that after the pectin was decomposed by the gut microbiota, increasing the reducing sugar level, the pectin digestion products were consumed, subsequently decreasing the reducing sugar level. The results also suggest that the gut microbiota composition differed between the donors because the ratios of reducing sugar production and consumption varied. Donor 3 might harbor many microorganisms that degrade pectin well, while donor 1 may harbor more microorganisms that utilize pectin degradation products. The carbohydrate contents of the sample media were determined by TLC (Fig. 1c). This confirmed that various fermentable sugars were present in the medium, but most were consumed at early stages. Galacturonic acid, the final degradation product of pectin, was produced after 6–12 h in all samples and was completely consumed at later time points. In the donor 3 sample, the production of galacturonic acid was faster than in the other samples, which confirmed that more pectin degradation products were produced. These results are consistent with the total carbohydrate and reducing sugar analyses described above, and indicates that the intestinal microbiota differed in each donor.

### Fecal microbial composition of each donor at baseline

To investigate the in vitro fermentation of pectin by the human fecal microbiota, we anaerobically incubated the feces of the three donors with 1% pectin for 18 h. The compositions of the fecal microbiota samples were observed at baseline (in raw fecal samples which were not mixed with medium or pectin) and after every 6 h of incubation time using 16S rRNA gene sequencing.

**Fig. 2:**
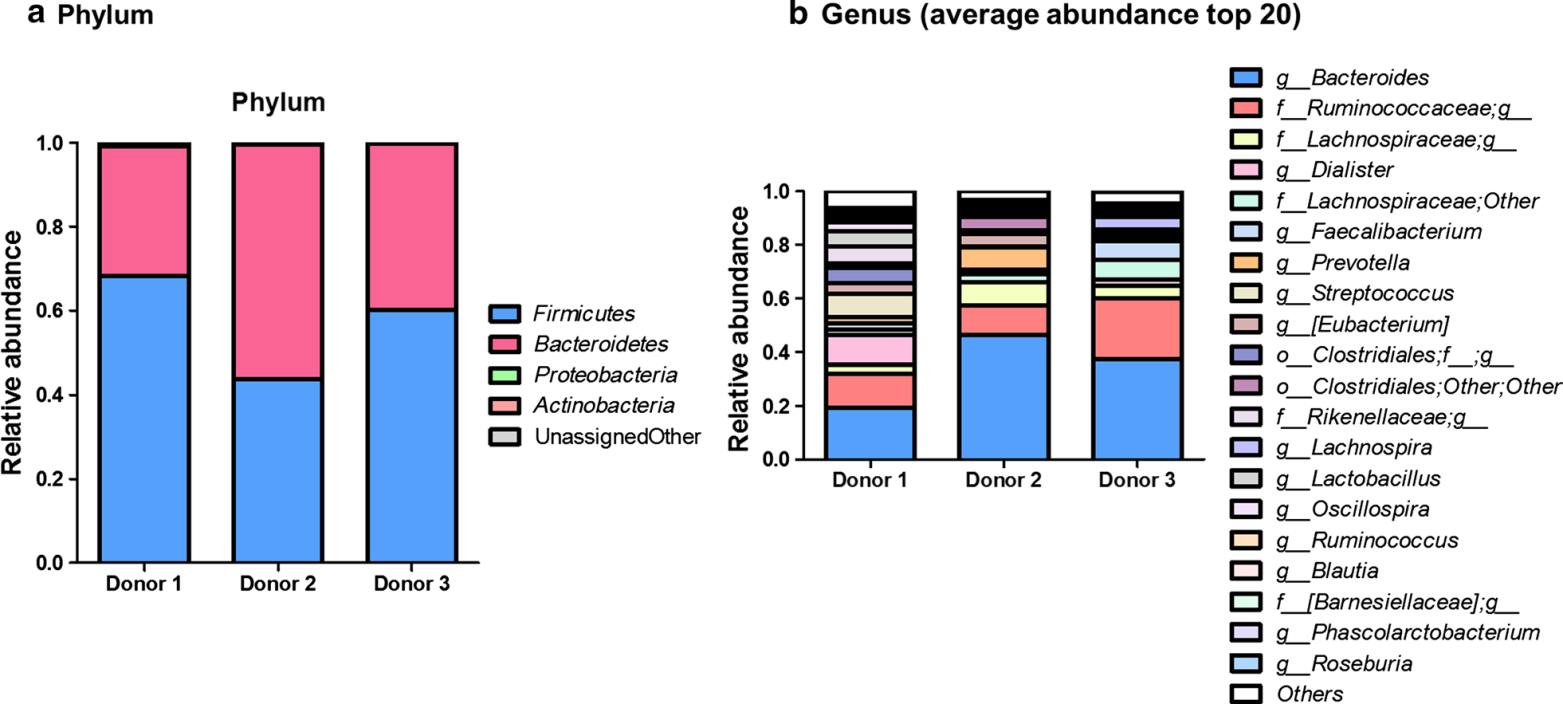
Baseline fecal microbial compositions of each donor, including the relative abundance of phyla (**a**) and the 20 most abundant genera (**b**)

We first examined the initial fecal microbiota compositions of each donor. At the phylum level, *Firmicutes* (57.39% of average relative abundance) and *Bacteroidetes* (42.11%) were dominant in all three donors, and *Proteobacteria* (0.26%) and *Actinobacteria* (0.02%) were also present in small proportions (Fig. 2a). The *Firmicutes*/*Bacteroidetes* ratios were higher in donor 1 (2.21 ratio) and donor 3 (1.52 ratio) compared to donor 2 (0.78 ratio). We next examined the top 20 most abundant genera in each baseline sample (Fig. 2b). Donors 1, 2, and 3 had 18, 12, and 11 genera comprising > 1% relative abundance, respectively. In all three donors, *Bacteroides* and *Ruminococcaceae* were the most abundant. The relative abundance of *Bacteroides* in donor 2 was much higher (46.34%) than that in donors 1 and 3 (19.20 and 37.39%, respectively). In addition, each donor displayed differences in bacterial abundance. For example, donor 3 harbored more *Lachnospiraceae* and *Faecalibacterium* than the others, while donor 1 and donor 2 harbored more *Streptococcus*, unclassified *Rikenellaceae*, *Lactobacillus*, *Lachnospiraceae*, and *Prevotella*.

### Changes in fecal microbial composition during pectin fermentation

**Fig. 3:**
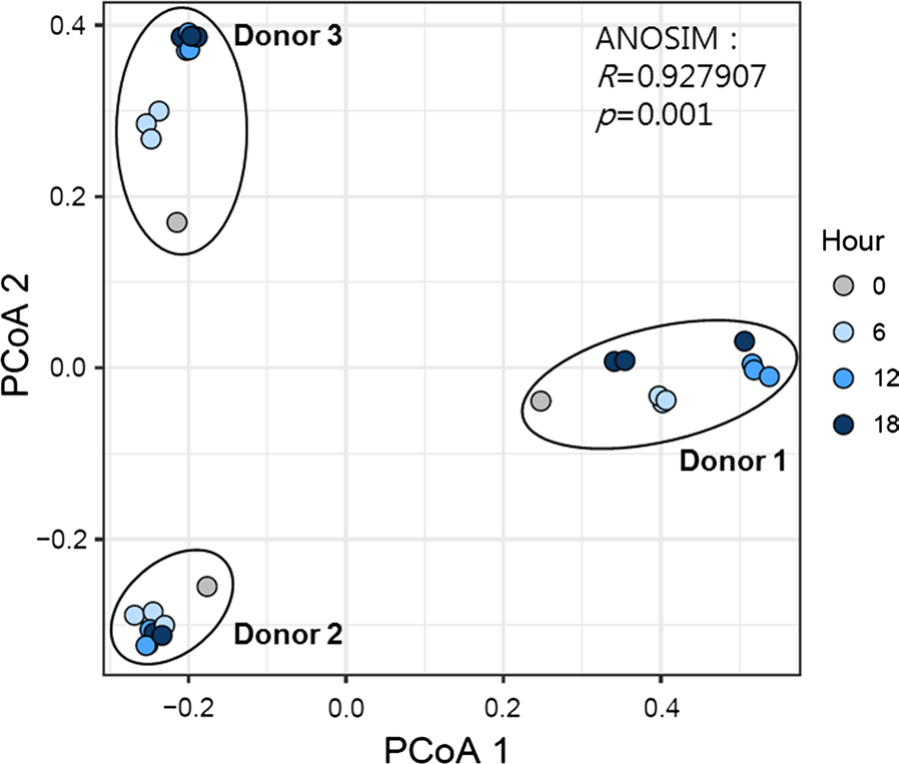
PCoA based on Bray–Curtis distances of fecal samples incubated with pectin for various incubation times

To evaluate the influence of pectin fermentation on the overall structure of the fecal microbiota, we conducted PCoA based on Bray–Curtis distances. Samples from each donor clustered separately from those of the other donors, showing that the donor had the greatest effect on microbiome composition, even after incubation with pectin for 18 h (Fig. 3). However, among samples from each donor, the microbial composition changed with increased incubation time. For donor 3, for example, the baseline sample was most similar to the sample after 6 h pectin incubation, followed by those taken at 12 and 18 h.

**Fig. 4:**
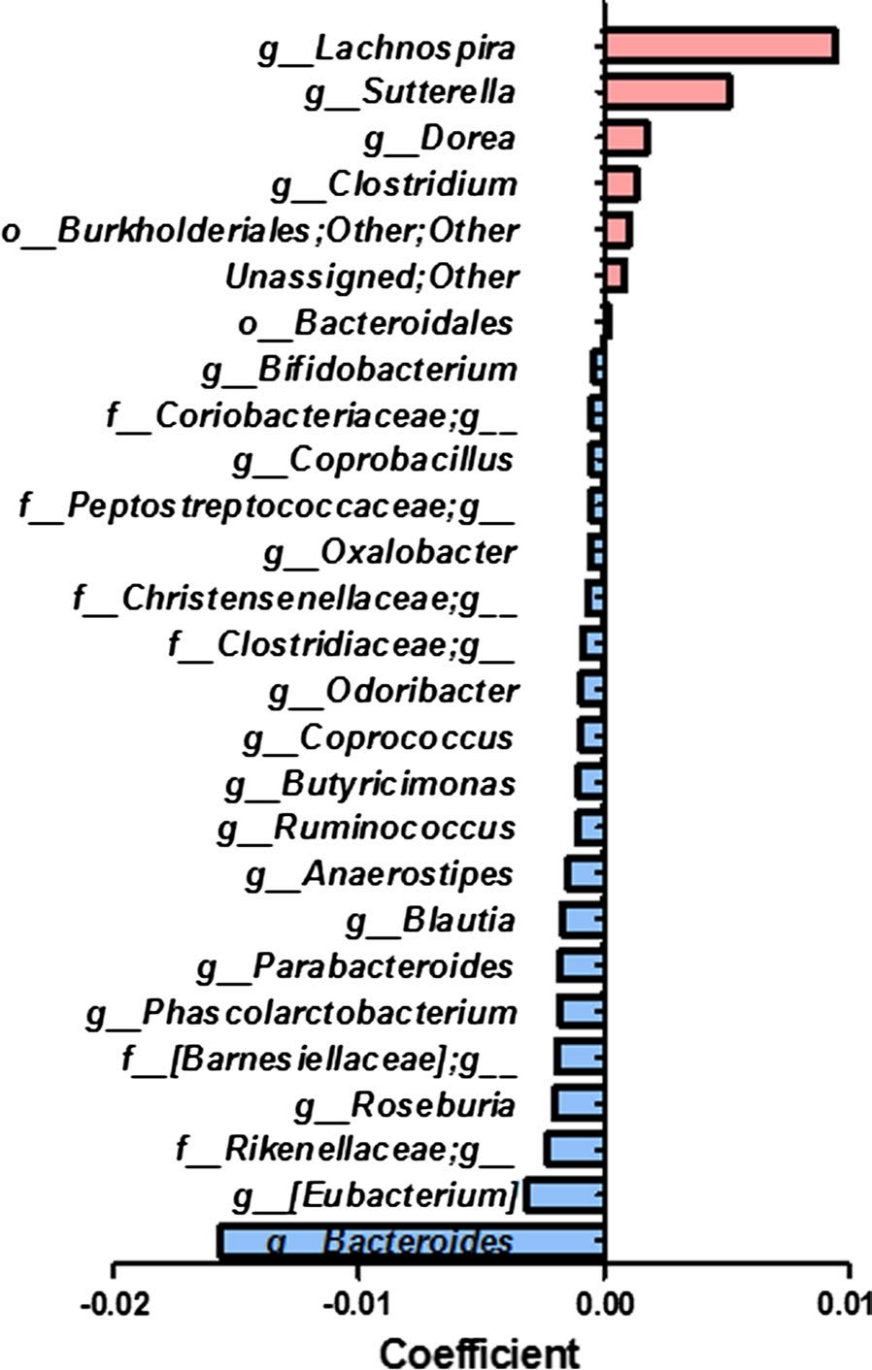
Significantly changed taxa according to pectin incubation time (q value < 0.1)

To identify microbes whose abundances significantly changed with increased incubation time, we explored the associations between bacterial abundance and incubation time by linear regression analysis. The levels of several specific bacteria were associated with time. For example, *Lachnospira*, *Sutterella*, *Dorea*, and *Clostridium* were significantly increased over time, while *Bacteroides* and *Roseburia* were significantly decreased (Fig. 4 and Additional file 1: Figure S1).

### SCFA formation during pectin fermentation

**Fig. 5:**
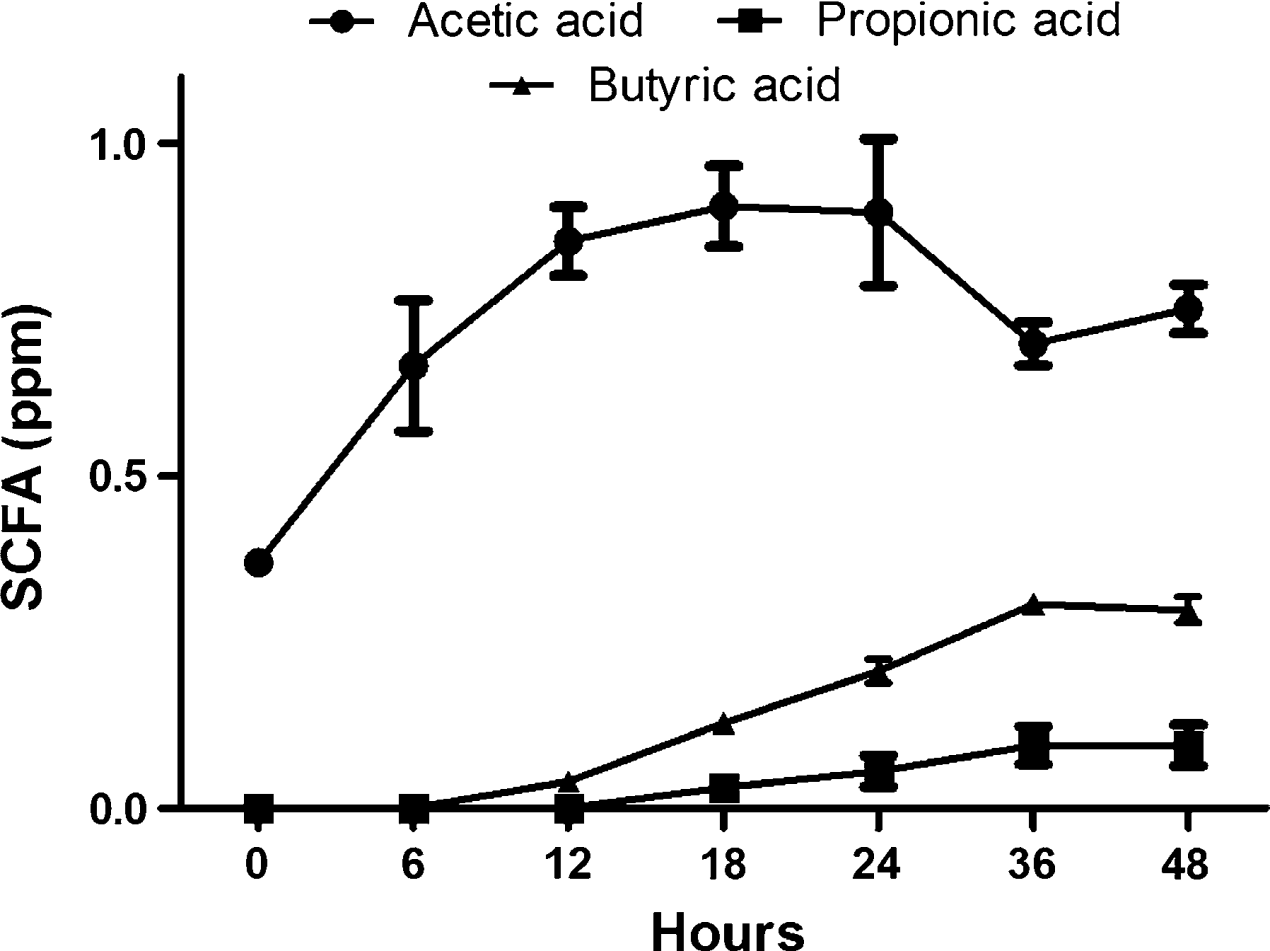
Average SCFA concentrations at various pectin incubation times

When we analyzed the SCFA changes in each sample every 6 h, we observed rapid increases in acetate after 6 h, which continuously increased up to 18 h, then rapidly decreased by 36 h. Propionate increased after 48 h, and while butyrate did not rapidly increase like acetate, it increased by approximately 28% by 48 h (Fig. 5).

## Discussion

The contributions of the gut microbiome to health and nutrition depend on its composition, which is affected by different factors, including lifestyle and diet (Conlon and Bird 2014). Gut microbiota composition can be changed by including indigestible carbohydrates (prebiotics) in one’s diet (Flint et al. 2012). Pectin is a prebiotic dietary fiber that affects the gut microbiota (Woods and Gorbach 2001). In this study, we investigated the utilization of pectin by the gut microbiota and analyzed microbiota composition changes with in vitro pectin fermentation through metagenomics analysis.

Pectin was mainly degraded between 0 and 18 h in all three donors, but the samples showed differences in their pectin degradation ability. These differences in substrate utility depend on the composition of the microbiome (Flint et al. 2008). When pectin was digested, galacturonic acid is produced (De Vries et al. 1982). Monosaccharide such as galacturonic acid is used as an energy source by bacteria and contributes to the development and maintenance of the gut microbiota (Zoetendal et al. 2012). In this study, the microbiota of donor 3 showed relatively high pectin utilization, and as expected, this sample contained higher baseline *Lachnospiraceae* and *Faecalibacterium* levels than in the other samples. *Lachnospiraceae* has been demonstrated to express carbohydrate-active and pectin-degrading enzymes (Biddle et al. 2013), and *Faecalibacterium* also has reported pectin degradation ability (Lopez-Siles et al. 2012). In addition, the sample from donor 2 consumed more galacturonic acid than the others once the reducing sugar level increased after 6 h. These results indicate that differences in the baseline levels of different microbiome components result in differences in pectin degradation and utilization in each donor.

We observed comprehensive overall gut microbiota changes after pectin fermentation in all three donors compared to their baseline compositions. We not only observed differences between each donor’s gut microbiota, but also changes with increased incubation time by PCoA. We assumed that specific gut microbiota were utilizing pectin as a substrate; for example, *Prevotella* and *Butyrivibrio* spp., which express pectinolytic enzymes (Marounek and Dušková 1999). With increased incubation time with pectin, we observed increases in *Lachnospira*, *Sutterella*, *Dorea*, and *Clostridium*. The *Clostridium coccoides* group (cluster XIVa) includes *Lachnospira*, *Dorea*, and *Clostridium* (Lopetuso et al. 2013). *Lachnospira* was the most increased and has been reported to use pectin as a substrate (Wojciechowicz et al. 1980) and *Lachnospira* plays a role that produced SCFA (Duvallet et al. 2017; Jones et al. 2014). Pectin degradation by *Lachnospira* affects the growth of other bacteria, including other saccharolytic bacteria, via cross-feeding (Salyers and Leedle 1983). Increases in *Dorea* and *Clostridium* may be due to *Lachnospira* cross-feeding. These results indicate that pectin promotes the presence of species in *Clostridium* cluster XIV, including *Lachnospira*, *Dorea*, and *Clostridium*. We also observed that some bacteria, including *Bacteroides*, which can utilize pectin (Gibson and Roberfroid 1995), decreased with increasing pectin incubation time, possibly due to environmental nutritional limitations caused by selective culture with pectin and carbohydrate consumption.

The production of SCFAs, including acetate, propionate, and butyrate, is affected by the composition of the gut microbiota, the utilization of carbohydrate substrates, and the gut environment, including the pH and other nutritional factors (Cummings and Englyst 1987; Yuan et al. 2006). Walker et al. (2005) reported that the production acetate and propionate increased at pH 6.5. *Lachnospira* produces the most pectin lyase at pH 6.1–6.3 (Silley 1986), and in pectin culture, mainly produces acetate and lactate (Dušková and Marounek 2001). The *Clostridium* genus is known to mainly produce butyrate (Rajilić-Stojanović and de Vos 2014). Based on this, we suggest that *Lachnospira* produced galacturonic acid through the digestion of pectin and acetate, and that this environment restrained the growth of *Bacteroides* by causing a mildly acidic pH. Produced galacturonic acid affected the composition of the gut microbiota, enhancing cluster XIVa species belonging to *Dorea* and *Clostridium*, which then produced butyrate (Duncan et al. 2007; Walker et al. 2005). We observed decreased acetate after 18 h of pectin incubation, indicating that not only galacturonic acid but also acetate was utilized by bacteria like *Faecalibacterium* to produce butyrate (Duncan et al. 2004; Ramirez-Farias et al. 2008).

This result suggests that acetate-producing bacteria like *Lachnospira* and *Faecalibacterium* caused increased butyrate levels via butyrate synthesis using acetate as a substrate (Khan et al. 2012; Rios-Covian et al. 2015). Based on this, it appears that pectin degradation results in a gut microbiota growth environment associated with the development of acetate and butyrate.

In conclusion, we demonstrate that in Korean individuals, pectin can change the gut microbiota by measuring total sugar levels and microbial composition over time. Pectin was completely degraded by the gut microbiota at 6, 12, and 18 h, and *Lachnospira* and *Faecalibacterium*, which can utilize pectin, were increased. Pectin-induced changes in the gut microbiota increased the formation of associated SCFAs from 6 h on, when pectin was decomposed. Therefore, we confirmed that pectin fermentation in gut microbiota samples from Korean individuals induced microbiota compositional changes. Increased pectin utilization and corresponding changes to gut microbiome composition may be beneficial to human health. Further analysis of the gut microbiomes of larger numbers of donors, in addition to experiments regarding cross-feeding and in vivo gut microbiota changes, would provide more accurate results.

## Additional file

**Additional file 1: Figure S1.** Several bacterial taxa significantly associated with pectin incubation time. Each donor is color-coded as indicated by the legend; (A) *Lachnospira*, (B) *Roseburia*, and (C) *Bacteroides*.

## Abbreviations

SCFAs: short chain fatty acids
CMR: bovine rumen fluid
CMRP: bovine rumen fluid pectin
DNS: 3,5-dinitrosalicylic acid
TLC: thin liquid chromatography
PCR: polymerase chain reaction
QIIME: the quantitative insights into microbial ecology
PCoA: principal coordinates analysis
IC: ion chromatography
USEARCH: ultra-fast sequence analysis
RDP: Ribosomal Database Project
