# Supplementary material for: The influence of in vitro pectin fermentation on the human fecal microbiome
Source: AMB Express. 2018 Jun 16;8:98. doi: 10.1186/s13568-018-0629-9 (PMC6004267; doi:10.1186/s13568-018-0629-9)
Supplement: Supplementary file 1 — Additional file 1: Figure S1. Several bacterial taxa significantly associated with pectin incubation time. Each donor is color-coded as indicated by the legend; (A) Lachnospira, (B) Roseburia, and (C) Bacteroides. [file 13568_2018_629_MOESM1_ESM.docx]

**AMB Express Journal**

**The influence of in vitro pectin fermentation on the human fecal microbiome**

So-Jung Bang^1a^, Gayoung Kim^1,a^, Mi Young Lim^2^, Eun-Ji Song^2,3^, Dong-Hyun Jung^1^, Jun-Seok Kum^4^, Young-Do Nam^2,3^, Cheon-Seok Park^1,^*, and Dong-Ho Seo^2,^*

*^1^Graduate School of Biotechnology and Institute of Life Science and Resources, Kyung Hee University, Yongin 17104, Republic of Korea*

*^2^Research Group of Gut Microbiome, Korea Food Research Institute, Wanju 55365, Republic of Korea*

*^3^Department of Food Biotechnology, Korea University of Science and Technology, Daejeon 34113, Republic of Korea*

*^4^Division of Strategic Food Research, Korea Food Research Institute, Wanju 55365, Republic of Korea*

^a^These authors contributed equally.

*Corresponding authors: E-mail: [sdh83@kfri.re.kr](mailto:sdh83@kfri.re.kr), cspark@khu.ac.kr

Dong-Ho Seo (Tel: +82 63-219-9385)

Cheon-Seok Park (Tel: +82 31-201-2631)


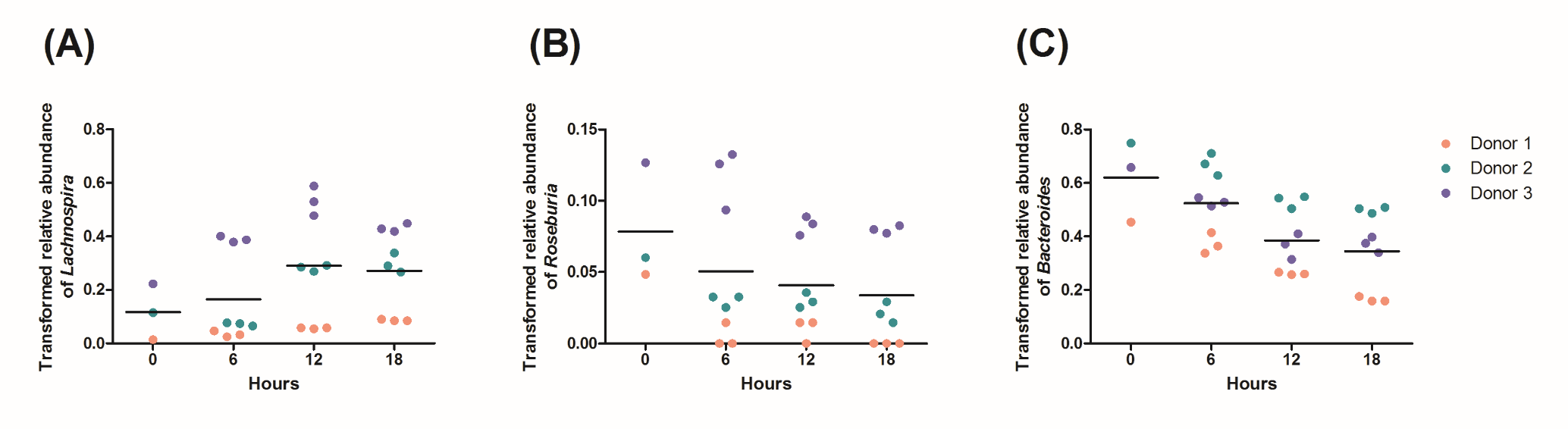


**Fig. S1 Several bacterial taxa significantly associated with pectin incubation time.** Each donor is color-coded as indicated by the legend**;** (A) *Lachnospira*, (B) *Roseburia*, and (C) *Bacteroides*
